# Machine Learning Approaches for Protein–Protein Interaction Hot Spot Prediction: Progress and Comparative Assessment

**DOI:** 10.3390/molecules23102535

**Published:** 2018-10-04

**Authors:** Siyu Liu, Chuyao Liu, Lei Deng

**Affiliations:** School of Software, Central South University, Changsha 410075, China; siyuliu@csu.edu.cn (S.L.); liuchuyao@csu.edu.cn (C.L.)

**Keywords:** hot spots, protein-protein interaction, machine learning, performance evaluation

## Abstract

Hot spots are the subset of interface residues that account for most of the binding free energy, and they play essential roles in the stability of protein binding. Effectively identifying which specific interface residues of protein–protein complexes form the hot spots is critical for understanding the principles of protein interactions, and it has broad application prospects in protein design and drug development. Experimental methods like alanine scanning mutagenesis are labor-intensive and time-consuming. At present, the experimentally measured hot spots are very limited. Hence, the use of computational approaches to predicting hot spots is becoming increasingly important. Here, we describe the basic concepts and recent advances of machine learning applications in inferring the protein–protein interaction hot spots, and assess the performance of widely used features, machine learning algorithms, and existing state-of-the-art approaches. We also discuss the challenges and future directions in the prediction of hot spots.

## 1. Introduction

Protein–protein interactions play critic roles in many physiological activities, such as gene replication, transcription, translation, and cell cycle regulation, signal transduction, immune response, etc. In order to understand and utilize these interactions, it is necessary to identify residues at the interface of the interaction [1]. Studies have shown that the protein interaction interface is usually large; a typical interaction interface is about 1200–2000 Å2, but only a few (<5%) of the residues called hot spots contribute to most of the binding free energy and play important roles in the stability of protein binding [2]. Deeper exploration of protein–protein interaction hot spots is critical to molecular recognition mechanisms and regulation, as well as a solid foundation for bioengineering such as protein engineering and drug design, and this solid foundation may still provide key clues for the identification of cancer-triggered genes in the future [3]. Experimental identification of hot spots is typically performed by alanine scanning mutagenesis. This process involves the mutation of a residue of interest to alanine in the bound and unbound state, and calculating the binding free energy changes (∆∆G). Widely used databases of experimental verified hot spots include the Alanine Scanning Energetics Database (ASEdb) [4], the Binding Interface Database (BID) [5], the Protein-protein Interaction Thermodynamic (PINT) [6], and the Structural database of kinetics and energetic of mutant protein interactions (SKEMPI) [7].

Analysis and exploration of the composition, structure and mechanism of hot spots is the basis for the development of prediction methods. Studies have shown that hot spots are not randomly composed of amino acids, and tryptophan (21%), arginine (13.3%), and tyrosine (12.3%) have the highest background probabilities of occurrence [2]. Most energy hot spots are tightly located in the protein's complemented pockets that are pre-organized in the unbound states [8]. These pockets show great complementarity with hot spots in shape and amino acid arrangement. Clackson and Wells proposed the O-ring theory [9], which reveals that hot spots are usually located in the center of protein interfaces, and they are surrounded by energetically less important residues that are shaped like an O-ring to block water molecule intrusion, and they provide a suitable solvent environment for functional hot spots. Li and Liu [10] proposed the double water exclusion hypothesis, which characterizes the topological organization of hot spots and their neighboring residues. These findings facilitate the development of computational methods to predict energy hot spots.

Existing hot spot prediction methods can be roughly divided into three types: knowledge-based methods, molecular simulation techniques, and machine learning methods [11]. The knowledge-based empirical function evaluates the change in binding free energy by reducing the empirical model obtained using experiments. The introduced molecular dynamics model uses alanine to perform fixed-point scanning by mutagenesis technology, and it detects the hot spot by detecting the change of binding energy (∆∆G) in the process of mutation to alanine. However, it is limited by factors such as the expensiveness of the experimental equipment, the long computing time it takes, and the limited number of hot spots tested. The machine learning approaches provides a more convenient way for hot spot prediction. 

**Figure 1:**
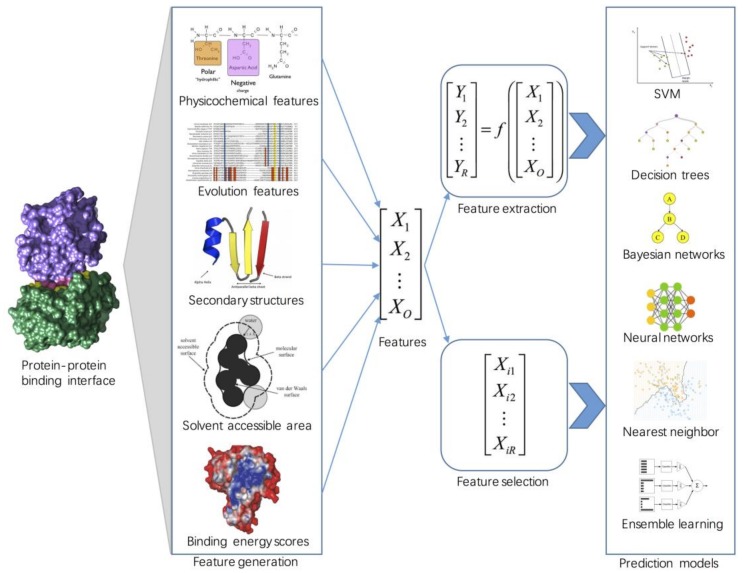
Overview of machine learning approaches to predicting protein–protein interaction hot spots. For the binding of interface residues in protein–protein interactions, a large number and variety of features are extracted from diverse data sources. Then feature extraction and feature selection approaches are used for dimensionality reduction. Finally, the machine learning-based prediction models are trained and applied to make predictions of hot spots.

Figure 1 shows the typical applications of machine learning in predicting protein-protein interaction hot spots. Usually, the input to the hot spot predictor is a target interface residue that is encoded by a variety of sequence, structural, and energy features. Dimensionality reduction (feature selection or feature extraction) is then used to remove the irrelevant and redundant information and to obtain a set of principal variables. Finally, predictive models are built using efficient machine learning algorithms. This paper focuses on machine learning-based methods and introduces some important issues that should be considered when adopting these approaches for hot spot prediction, including feature generation, dimensionality reduction, and algorithm design. More importantly, we generate a benchmark dataset and an independent dataset to investigate the performance of widely used biological features and classical machine learning algorithms. We also perform an independent test to evaluate the performance of state-of-the-art hot spot prediction approaches. The datasets, features, and results of this study are freely available at http://denglab.org/pphot_review/.

## 2. Feature Engineering

The steps of using machine learning to predict hot spots usually include data preparation, feature engineering, choosing a machine learning model, a training and testing model, and predicting the output. Feature engineering is a crucial step for developing effective hot spot prediction approaches, since the features have a significant impact on the prediction performance. Often, a large number of features or attributes are collected from the protein sequence, structure, and energy data. Dimensionality reduction approaches are used to obtain the most effective features for future classification tasks.

### 2.1. Sequence-Based Features

Protein sequence features, including the physicochemical properties of amino acids, evolutionary information in terms of evolutionary conservation score and position-specific scoring matrix (PSSM), and other sequence descriptors, have been widely used in computational biology [12,13]. Physicochemical features (e.g. hydrophobicity, hydrophilicity, polarity and average accessible surface area) from the AAindex1 database [14] are extracted to predict hot spots [15,16]. Position-specific scoring matrices (PSSMs) are a commonly used sequence feature that can be obtained from NCBI non-redundant databases via PSI-BLAST [17]. Several studies [11,18,19] have used PSSMs for hot spot prediction. The local structural entropy (LSE) [20] mainly describes the degree of consistency of protein sequences. It has also been proven to be useful in the prediction of hot spots [21]. The evolutionary conservation score is calculated using multiple sequence alignments (MSAs) and a phylogenetic tree [22]. Higa et al. [23] incorporated a conservation score, an evolutionary profile, and other structural features to predict binding hot spot residues. Shingate et al. developed a computational approach named ECMIS [24] to identify hotspots using a conservation score, a mass index score, and an energy scoring scheme.

### 2.2. Structure-Based Features

Protein tertiary structure refers to the folding arrangement of amino acids in three dimensions, which can help to understand the function of proteins at the molecular level. Incorporating structural features can better apply the spatial structure features of proteins to hot spot prediction, and generally obtains better results than sequence-based features. The solvent accessible surface area (ASA) is defined as the locus of the center of the virtual solvent molecule as it rolls over the surface of the protein, and it is usually calculated by DSSP (Definition of Secondary Structure of Proteins) [25] and Naccess [26]. ASA-related features are widely used in protein–protein interaction interfaces and hot spot prediction [11,12,27,28]. Biochemical contacts, including atom contacts, residue contacts, hydrogen bonds, and salt bridges, are also important structural features for predicting hot spots [29,30,31]. The four-body statistical pseudo-potential (FBS2P) is calculated based on the Delaunay triangulation of proteins [32,33], and it has been used in PredHS [11,34].

### 2.3. Energy-Based Features

Energy features have been applied to hot spot prediction in recent studies. Kortemme et al. [35] used a linear combination of a Lennard–Jones potential, an implicit solvation model, an orientation-dependent hydrogen-bonding potential, and an estimate of unfolded reference state energies for the prediction of energetically important residues. Tuncbag et al. [27,36] applied statistical inter-residue pair potentials to improve the accuracy of hot spot prediction. Lise et al. [37,38] calculated Van der Waals potentials, solvation energy, side-chain intermolecular energies, environment intermolecular energies, and side-chain intramolecular energies to the predictions of hot spot residues. Deng et al. [11] incorporated side-chain energy, residue energy, interface propensity, and two combined energy scores calculated by ENDES [32,39].

### 2.4. Feature Selection

Feature selection can provide a deeper insight into the underlying means that generate the data, avoid overfitting, and improve the prediction performance [40]. Typical feature selection algorithms include the F-score [41], random forest [42], support vector machines–recursive feature elimination (SVM-RFE) [43], minimum redundancy maximum relevance (mRMR) [44,45], and maximum relevance maximum distance (MRMD) [46]. Several feature selection approaches have been used for hot spot prediction. APIS [28] used the F-score to select relevant features. MINERVA [30] used a decision tree to select useful features. Wang et al. [47] and Moreira et al. [19] used random forest to predict hot spots. PredHS [12] combined random forest and sequential backward elimination algorithms to select optimal features for predicting hot spots. Qiao et al. [48] developed a hybrid feature selection strategy which combines the F-score, mRMR, and the decision tree.

### 2.5. Feature Extraction

Feature extraction is another dimensional reduction approach in machine learning applications. Principal component analysis (PCA) [49,50] and linear discriminant analysis (LDA) [51] are two commonly used feature extraction techniques. PCA works by establishing an orthogonal transformation of the data to convert a set of possible correlated variables into a set of linearly-uncorrelated ones, the so-called principal components. Melo et al. [18] applied PCA to reduce the dimensionality of a high-dimensional dataset (79 features), and improved hot spot prediction. Moreira et al. [19] used PCA to generate different datasets (PCA, PCAUp and PCADown) and evaluated the performance in hot spot prediction.

## 3. Machine Learning Approaches for Hot Spot Prediction

In addition to selecting effective features or feature combinations, using appropriate machine learning methods can also play an important role in improving the performance of hot spot prediction. Machine learning methods, such as nearest neighbor [52], support vector machines [53], decision trees [54], Bayesian networks [55], neural networks [56], and ensemble learning [57], have been widely used in protein–protein interaction hot spots prediction in recent years. Table 1 summarizes the existing machine learning-based methods for hot spot identification.

### 3.1. Nearest Neighbor

The nearest neighbor algorithm [52] is an instance-based lazy learning method, and one of the simplest understandings of machine learning algorithms. Hu et al. [58] proposed a protein sequence-based model, in which the classifier is implemented by the improved IBK (Instance-based k means) algorithm of the k-nearest neighbors, which overcomes the shortcomings of the recent neighbor algorithm, which is sensitive to some data. Jiang et al. [16] also proposed a sequence-based model, using the IBK algorithm to obtain a better random projection set through the training set.

### 3.2. Support Vector Machines

SVM [53] is the most widely used machine learning method. It establishes the optimal hyperplane in a high-dimensional feature space to ensure the classification risk by ensuring the minimum structural risk. It has the advantages of high efficiency and high accuracy, but it also has shortcomings such as input data requiring labels and only being suitable for two types of classification problems. Several hot spot prediction models are built using SVM [3,11,28,30,48,59].

Cho et al. [30] proposed MINERVA, which used 54 features of structure, sequence, and molecular interaction, and selected the top three best features using a decision tree. They used a support vector machine to create a predictive model of protein–protein interaction hot spots. Xia et al. [28] carefully studied 62 sequence and structure features, and used the F-score to remove redundant features. The APIS predictor has been developed to identify hot spots using SVM. The experimental results show that APIS can identify more hot spots than traditional hot spot prediction methods. Zhu et al. [60] built two hot spot prediction models (KFC2a and KFC2b) using support vector machines. PredHS [11] used 38 optimal selected features to train SVM models, and it demonstrated a significant improvement in predictive performance. Ye et al. [59] selected the optimal 58-dimensional feature subset containing 10 network and micro-environment features by a random forest algorithm, and then applied the feature subset and support vector machine to construct a hot spot prediction model. HEP [3] used 108 sequences, structures, and domain features, and selected two highest-ranking features using a two-step feature selection method. The final prediction model was constructed by using the support vector machine. Lise et al. [37] and Higa et al. [23] also incorporated the SVM classifier to predict hot spots.

### 3.3. Decision Trees

As a widely used supervised learning method, the decision tree [54] represents a mapping relationship between features and tags in the predictive model. Each branch is a predicted output; a category represented by each leaf node. One of the ways in which decision-making stops branching is pruning, which helps to achieve tree balance. In addition to the advantages of easy understanding and simple data preparation, the decision tree can not avoid the disadvantages of increasing the error rate of the category and making it difficult to predict continuous fields. The classic KFC (knowledge-based FADE and contacts) method [31] is a combination of two decision tree models, K-FADE and K-CON. The machine learning algorithm C5.0 [61] was used to search for patterns within the training data, and to generate a learned decision tree that predicts the hot spot residues within the protein–protein complexes.

### 3.4. Bayesian Networks

As an extension of the Bayesian method [55], the Bayesian networks [62,63] magnify the independent hypothesis of each variable on the premise hypothesis compared to the naive Bayesian foundation [64], which assumes that each variable is discrete. This mathematical model based on probabilistic reasoning, which is based on the combination of the Bayesian principle and graph theory, has good performance in solving the problem of strong correlation, but its shortcoming is mainly reflected in its inability to filter variables. The PCRPi [65] method combined three main sources of information, namely the energy, structure, and evolutionary determinants of the Bayesian network (BN). The Bayesian network toolbox for MatLab (BNT) was used to implement BNs, and the R package 'Deal' was used to learn the structure of expert BNs. A large number of experiments have proven that PCRPi can provide consistent and accurate prediction results in hot spot prediction. Most importantly, PCRPi can handle some of the missing protein data, as well as unreliable conditions.

### 3.5. Neural Networks

Artificial neural networks (ANN) [56] simulate human intuitive thinking, which can form distributed storage of data and parallel collaborative processing. Here, each node represents a particular output function, and the connection between the nodes represents the weighted value of the signal. The development of artificial neural networks has shown excellent and intelligent features in pattern recognition, and biology and medicine. Ofran and Rost [66] predicted that a residue is a hot spot of interaction from the sequence of a single protein, and it does not need to know the interacting partner. They trained standard feed-forward neural networks with back-propagation and momentum terms on windows of nine consecutive residues.

### 3.6. Ensemble Learning

Ensemble methods are machine learning algorithms that combine multiple classifiers into one predictive model to obtain better predictive performance. Many ensemble algorithms exist, including random forest [67], AdaBoost [68], gradient tree boosting [69], xgboost [70], etc.

Wang et al. [71] proposed a novel random forest (RF) model to effectively integrate hybrid features, including a wide range of information on the target residue and its spatially neighboring residues, for predicting hot spots in protein interfaces. Huang et al. [72] used SMOTE [73] to process unbalanced data, and applied AdaBoost to predict protein hot spots. Deng et al. [11] proposed an ensemble model (PredHS-Ensemble), which uses an ensemble of n classifiers and a decision fusion technique on the training datasets. An asymmetric bootstrap resampling approach is adopted to generate subsets. It performs random sampling with replacement only on the majority class so that its size is equal to the number of minority samples, and keeps the entire minority samples in all subsets.

## 4. Comparative Assessment

### 4.1. Datasets

We constructed a benchmark dataset from four databases, including Alanine Scanning Energetics (ASEdb) [4], SKEMPI database [7], Assi et al.'s Ab+ data [65] and Petukh et al.'s Alexov_sDB [74]. We combined the alanine-mutated data from the four databases, and excluded the proteins existing in the BID dataset [5]. We used CD-HIT [75] to remove the redundant proteins and obtained a benchmark of 34 protein complexes, which contained 313 mutated interface residues. The interface residues were defined as hot spots with ∆∆G >= 2.0 kcal/mol, and the others were defined as non-hot spots. As a result, the benchmark (HB34) contained 133 hot spots residues and 180 non-hot spot residues.

We also generated an independent test dataset from the BID database [5]. Only “strong” mutations in the BID database were defined as hot spots, and others were non-hot spots. The proteins in this independent test set were non-homologous to those proteins in the above training dataset. The test dataset (BID18) was a collection of 18 complexes containing 127 alanine-mutated residues, where 39 interface residues were hot spots.

### 4.2. Performance Measures

To quantify how correct are the predictions made by an algorithm, we performed 50 times 10-fold cross-validation on the training benchmark dataset and computed commonly used measures, including specificity (SPE), precision (PRE), sensitivity (SEN), accuracy (ACC), F1-score (F1), and Matthews correlation coefficient (MCC).

(1) $$SPE=\frac{TN}{TN+FP}$$

(2) $$PRE=\frac{TP}{TP+FP}$$

(3) $$SEN=\frac{TP}{TP+FN}$$

(4) $$ACC=\frac{TP+TN}{TP+TN+FP+FN}$$

(5) $$F1=\frac{2\times SEN\times PRE}{SEN+PRE}$$

(6) $$MCC=\frac{TP\times TN-FP\times FN}{\sqrt{\left(TP+FP)(TP+FN)(TN+FP)(TN+FN\right)}}$$

where TP, TN, FP, and FN represent the numbers of true positive, true negative, false positive, and false negative residues in the prediction, respectively. We also calculated the area under the receiver operating characteristic curve (AUC) to evaluate the overall prediction performance.

### 4.3. Performance Evaluation of Different Features

As described in Section 2, a wide range of sequence, structures, and energy-based features have been utilized for hot spot prediction. Here we only evaluated five categories of representative features, including physicochemical features (12 features) [14], position-specific score matrix (PSSM) (20 features) [17], blocks substitution matrix (Blosum62) (20 features) [76], solvent accessible area (ASA) (six features) [77], and solvent exposure (seven features) [78]. Eleven physicochemical features (hydrophobicity, hydrophilicity, polarity, polarizability, propensities, average accessible surface area, number of atoms, number of electrostatic charges, number of potential hydrogen bonds, molecular mass, and electron–ion interaction pseudopotential) were obtained from the AAindex database [14], and pseudo hydrophobicity (PSHP) is defined in HEP [3]. PSSM profiles were calculated using PSI-BLAST [17], searching against the NCBI non-redundant database with parameters j = 3 and e = 0.001. The relative frequencies of amino acids and their substitution probabilities were computed using Blosum62 [76]. ASA features were calculated using DSSP [25]. Exposure features were computed using hsexpo [78], including HSEAU (number of C_α_ atoms in the upper sphere), HEAD (number of C_α_ atoms in the lower sphere), HSEBU (the number of C_β_ atoms in the upper sphere), HSEBD (the number of C_β_ atoms in the lower half sphere), CN (coordination number), RD (residue depth), and RDa (Cα atom depth). We also combined the five categories of features (Combined) to investigate whether fusion features would improve the performance.

We used three classical algorithms, including support vector machine (SVM) [53], random forest (RF) [67], and gradient tree boosting (GTB) [69], to build the classifiers. To compare the performance of these features more fairly, the 10-fold cross-validation procedure was repeated 50 times, and the average performance was calculated. As shown in Table 2, structural features (ASA and solvent exposure) performed significantly better than sequence features (physicochemical features, PSSM and blocks substitution matrix). For SVM models, the F1 score, MCC, and AUC of the sequence characteristics were 0.51~0.52, 0.17~0.20, and 0.56~0.63, respectively, while these measures of the structural features were 0.63, 0.33~0.36, and 0.72~0.73, respectively. Similar results were obtained on the RF and GTB models. The ASA-related features performed better than the other four categories of features (physicochemical features, PSSM, blocks substitution matrix, and solvent exposure features) on all of the three machine learning models. Among the three machine learning algorithms (SVM, RF and GTB), GTB had the best performance for single or combined features.

We also evaluated the performance of the feature combinations. The results are shown in Table 3. Due to the large number of pair combinations, only the results of using the GTB classifier are listed. In general, combining two types of features was better than using a single type of feature. Among these pair combinations, combining ASA related features and PSSM (ASA+PSSM) achieved the best predictive performance, with AUC and F1 scores of 0.761 and 0.663, respectively. As expected, the combination of all features showed the best predictive performance with sensitivity = 0.727, precision = 0.656, F1 = 0.681, and AUC = 0.787 when using GTB as the modeling algorithm. The results indicated that a combination of sequence and structural features can boost the performance of prediction. 

To further evaluate the performance of various features, we used the BID18 dataset for independent test. The results are shown in Table 4. The overall performance of the independent test was worse than the 10-fold cross-validation (Table 2). Among the five categories of features, ASA related features had the best performance, but solvent exposure performed similar or worse than the sequence features in hot spot prediction. This indicates that structural features are not always better than sequence features. Like 10-fold cross-validation, the combination of all the sequence and structural features were significantly better than the individual features. Combining more effective features may further improve the prediction performance.

**Figure 2:**
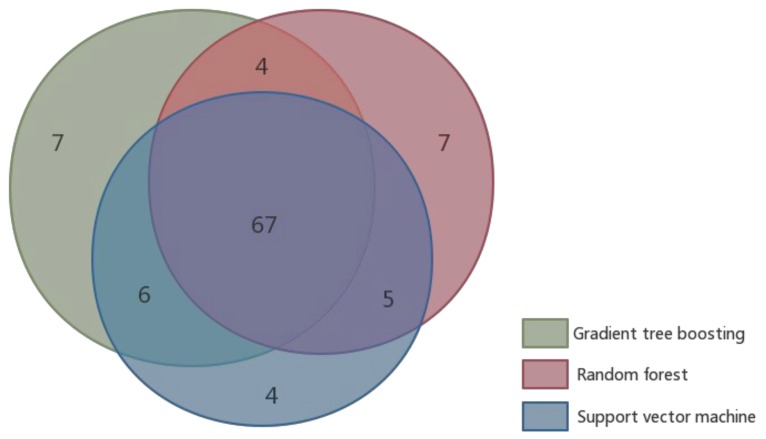
A Venn diagram showing the number of correctly predicted residues from the three machine learning algorithms for the independent dataset (BID18).

We summarized the numbers of residues that were correctly predicted using the three machine learning approaches (SVM, RF, and GTB) with combined features on the independent dataset (Figure 2). The results predicted by the three machine learning methods were mostly the same. 67 out of 127 residues, of which 26 were hot spots, and 41 were non-hot spots, were correctly predicted by all of the three machine learning algorithms. A small number of residues could only be predicted by one or two machine learning algorithms. For example, there were seven residues that could only be correctly predicted by GTB, and there were six residues that could only be correctly predicted by GTB and SVM. The results were consistent with our expectations, because these machine learning methods use the same features. The number of true positives (TP), true negatives (TN), false positives (FP) and false negatives (FN) of the three machine learning methods are shown in Table 5. Some proteins (e.g. 1FAK_T, 1G3I_A, and 1GL4_A) were well predicted, but some (e.g. 1DVA_H and 1JPP_B) were hard to predict. One of the possible reasons is that the training set had only a small number of experiment-determined hot spots.

### 4.4. Performance Comparison of Existing Hot Spot Prediction Methods

As summarized in Table 1, a variety of existing hot spot prediction approaches have been proposed in the past few years. Comparing the performance of these published methods is difficult, mainly because the heterogeneity of the datasets that are employed to benchmark the methods, and sometimes the difficulty of obtaining the methods themselves. Here, we evaluate some widely used methods that are easier to implement, or that have a web server using the BID18 dataset. These methods include HEP [3], PredHS [11], iPPHOT [48], KFC2 [60], PCRPi [65,79], MINERVA [30], APIS [28], KFC [31], Robetta [80], and FOLDEF [81]. The results are shown in Table 6. Regarding the overall performance, HEP had the highest F1 score of 0.70. The F1 score is a robust measure that estimates the relationship between the precision and the sensitivity; hence, HEP has a better balance between precision and sensitivity rates. PredHS-SVM achieved the best precision (PRE = 0.79). High-precision is very useful since the costs of false positives are high in practical application. Although HEP and PredHS-SVM performed well in the independent test, the overall performance was still relatively low, and there is still much room for improvement.

## 5. Discussion

Predicting protein–protein binding hot spots on protein interfaces will become increasingly crucial as reliable identification of protein binding hot spots has broad applications in computational protein design and drug discovery. In this paper, we present a comprehensive survey on machine learning approaches for protein–protein interaction hot spot prediction. These approaches are categorized based on the features that they utilize, and different machine learning algorithms. We evaluate the performance of widely used features and machine learning algorithms using a 10-fold cross-validation and independent test. We also perform independent test for the existing state-of-the-art approaches. The evaluation results show that as more and more features are discovered, the application of new machine learning methods, and the field of computational identifying hot spots has made great progress in recent years. Although there has been significant progress, there are many difficulties and much room for improvement in hot spot prediction. Challenges and future directions are summarized as follows:

(1) Hot spots are mainly discovered through biological experiments, lacking mature theoretical support and unified identification standards. Although the O-ring theory [9] with great influence explains the arrangement relationship between energy hot spots and surrounding residues well, it still has much controversy; the change of free energy (∆∆G) is usually used to discriminate energy hot spots, but different articles use different thresholds under different conditions, and they lack uniform standards.
(2) Systematic mutagenesis experiments are currently expensive and time-consuming to perform; the experimental data of energy hot spots are very limited, resulting in a lack of large benchmark datasets. As we observed in this study, supervised learning methods, especially GTB, have achieved good results, but the performance of each 10-fold cross-validation varies on repetition. Alternatively, semi-supervised learning and transductive inference approaches can be used to take advantage of the large number of unlabeled data to further improve the predictive performance.
(3) Due to the small number of samples and the large number of features in hot spot prediction, machine learning methods are easy to overfit. Improved feature extraction methods and feature selection approaches can help avoid overfitting. At the same time, the number of hot spots is far less than the number of non-hot spots, leading to the so-called imbalance problem. It is necessary to design effective algorithms (e.g. ensemble learning) to solve this problem.
(4) The characteristics of accurately identifying energy hot spots have not been well discovered, and no single feature can fully identify energy hot spots from the interface residues. This requires finding new and effective features, and studying the effects of combining different categories of features. For example, most existing machine learning hot spot predictors use statistical sequence and structural information to encode input feature vectors, but the spatial arrangement of residues has not been well exploited.
(5) Molecular dynamics simulation and molecular docking techniques can simulate the changes in binding free energy before and after alanine mutation. A promising future direction is developing effective ways to combine computational docking with machine learning methods, which has the potential to dramatically boost hot spot predictions.

## Figures and Tables

**Table 1:** Summary of machine learning classification methods for protein–protein interaction hot spot prediction.

Classification Methods	Description	References
Nearest neighbor	The model consists of 83 classifiers using the IBk algorithm, where instances are encoded by sequence properties.	Hu et al. [58]
	Training the IBk classifier through the training dataset to obtain several better random projections and then applying them to the test dataset.	Jiang et al. [16]
Support vector machine	The decision tree is used to perform feature selection and the SVM is applied to create a predictive model.	Cho et al. [30]
	F-score is used to remove redundant and irrelevant features, and SVM is used to train the model.	Xia et al. [28]
	Proposed two new models of KFC through SVM training	Darnell et al. [31]
	The two-step feature selection method is used to select 38 optimal features, and then the SVM method is used to establish the prediction model.	Deng et al. [11]
	The random forest algorithm is used to select the optimal 58 features, and then the SVM algorithm is used to train the model.	Ye et al. [59]
	Use the two-step selection method to select the two best features, and then use the SVM algorithm to build the classifier.	Xia et al. [3]
	When the interface area is unknown, it is also very effective to use this method.	Qian et al. [48]
Decision trees	Formed by a combination of two decision tree models, K-FADE and K-CON.	Darnell et al. [31]
Bayesian networks	Can handle some of the missing protein data, as well as unreliable conditions.	Assi et al. [65]
Neural networks	Does not need to know the interacting partner.	Ofran and Rost [66]
Ensemble learning	The mRMR algorithm is used to select features, SMOTE is used to handle the unbalanced data, and finally AdaBoost is used to make prediction.	Huang and Zhang [72]
	Random forest (RF) is used to effectively integrate hybrid features.	Wang et al. [71]
	Bootstrap resampling approaches and decision fusion techniques are used to train and integrate sub-classifiers.	Deng et al. [11]

**Table 2:** Performance comparison of different features on the benchmark dataset (HB34).

Methods	Features	SPE	SEN	PRE	ACC	F1	MCC	AUC
SVM	Physicochemical	0.672	0.521	0.545	0.608	0.520	0.196	0.566
	PSSM	0.696	0.504	0.553	0.614	0.515	0.204	0.634
	Blocks substitution matrix	0.644	0.522	0.529	0.594	0.511	0.170	0.595
	ASA	0.677	0.688	0.612	0.660	0.638	0.362	0.737
	Solvent exposure	0.609	0.726	0.580	0.658	0.635	0.339	0.724
	Combined	0.711	0.638	0.684	0.699	0.652	0.393	0.757
RF	Physicochemical	0.624	0.549	0.521	0.592	0.522	0.174	0.635
	PSSM	0.682	0.561	0.567	0.632	0.555	0.244	0.648
	Blocks substitution matrix	0.620	0.550	0.521	0.590	0.523	0.17	0.632
	ASA	0.722	0.587	0.614	0.664	0.589	0.312	0.696
	Solvent exposure	0.682	0.552	0.565	0.626	0.549	0.236	0.669
	Combined	0.756	0.656	0.624	0.699	0.631	0.384	0.766
GTB	Physicochemical	0.587	0.586	0.514	0.586	0.535	0.173	0.635
	PSSM	0.612	0.641	0.550	0.624	0.584	0.251	0.669
	Blocks substitution matrix	0.591	0.588	0.517	0.591	0.540	0.179	0.635
	ASA	0.665	0.648	0.588	0.658	0.608	0.310	0.693
	Solvent exposure	0.624	0.639	0.558	0.631	0.587	0.261	0.669
	Combined	0.717	0.656	0.727	0.719	0.681	0.439	0.787

**Table 3:** Performance comparison of feature combinations on the benchmark dataset (HB34) using GTB.

Methods	Features	SPE	SEN	PRE	ACC	F1	MCC	AUC
**GTB**	ASA + PSSM	0.708	0.705	0.642	0.707	0.663	0.410	0.761
	PSSM + Solvent exposure	0.671	0.718	0.617	0.691	0.656	0.385	0.760
	Blosum62 + Solvent exposure	0.664	0.699	0.606	0.679	0.640	0.359	0.734
	ASA + Solvent exposure	0.674	0.695	0.612	0.683	0.642	0.366	0.728
	Phy+Solvent exposure	0.664	0.696	0.605	0.677	0.639	0.357	0.728
	ASA + Blosum62	0.658	0.651	0.585	0.656	0.608	0.307	0.718
	ASA + Phy	0.669	0.644	0.590	0.658	0.607	0.311	0.717
	Phy + PSSM	0.629	0.650	0.566	0.638	0.597	0.277	0.683
	PSSM + Blosum62	0.619	0.655	0.560	0.635	0.595	0.271	0.679
	Phy + Blosum62	0.593	0.590	0.520	0.592	0.541	0.183	0.639
	Combined (all features)	0.717	0.656	0.727	0.719	0.681	0.439	0.787

**Table 4:** Performance comparison of different features on the independent test dataset (BID18).

Methods	Features	SPE	SEN	PRE	ACC	F1	MCC	AUC
SVM	Physicochemical	0.577	0.393	0.597	0.583	0.472	0.162	0.634
	PSSM	0.675	0.438	0.561	0.640	0.491	0.223	0.663
	Blocks substitution matrix	0.626	0.435	0.632	0.628	0.512	0.242	0.661
	ASA	0.597	0.446	0.716	0.634	0.549	0.290	0.693
	Solvent exposure	0.642	0.403	0.532	0.608	0.456	0.167	0.617
	Combined	0.569	0.464	0.832	0.650	0.586	0.353	0.732
RF	Physicochemical	0.632	0.414	0.576	0.614	0.479	0.196	0.624
	PSSM	0.703	0.417	0.474	0.632	0.443	0.171	0.616
	Blocks substitution matrix	0.62	0.408	0.575	0.607	0.474	0.185	0.627
	ASA	0.604	0.437	0.686	0.629	0.534	0.268	0.679
	Solvent exposure	0.59	0.402	0.612	0.597	0.484	0.188	0.64
	Combined	0.612	0.466	0.753	0.656	0.575	0.338	0.758
GTB	Physicochemical	0.531	0.384	0.643	0.566	0.478	0.163	0.625
	PSSM	0.681	0.416	0.506	0.627	0.456	0.178	0.638
	Blocks substitution matrix	0.580	0.400	0.617	0.592	0.480	0.184	0.624
	ASA	0.585	0.437	0.718	0.626	0.543	0.280	0.679
	Solvent exposure	0.592	0.389	0.579	0.588	0.465	0.159	0.646
	Combined	0.621	0.476	0.766	0.666	0.597	0.378	0.769

**Table 5:** Detailed prediction results for each protein on the independent test dataset (BID18).

PDB ID	GTB				RF					SVM			
	TP	FP	TN	FN		TP	FP	TN	FN	TP	FP	TN	FN
1CDL_A	1	1	1	0		1	1	1	0	1	1	1	0
1CDL_E	5	3	1	0		5	1	3	0	5	3	1	0
1DVA_H	0	4	7	1		0	4	7	1	0	4	7	1
1DVA_X	3	3	4	1		4	2	5	0	4	3	4	0
1DX5_N	1	1	13	2		1	2	12	2	2	3	12	0
1EBP_A	3	0	1	0		3	0	1	0	3	0	1	0
1EBP_C	1	3	1	0		1	1	3	0	1	0	4	0
1ES7_A	1	3	0	0		0	3	0	1	1	3	0	0
1FAK_T	2	5	14	0		2	5	14	0	2	7	12	0
1FE8_A	0	3	1	0		0	3	1	0	0	3	1	0
1FOE_B	1	0	1	0		1	0	1	0	0	0	1	1
1G3I_A	6	0	0	0		5	0	0	1	6	0	0	0
1GL4_A	4	1	1	1		3	2	0	2	3	1	1	2
1IHB_B	0	2	2	0		0	2	2	0	0	2	2	0
1JAT_A	1	0	0	0		0	0	0	1	0	0	0	1
1JAT_B	1	0	0	0		1	0	0	0	1	0	0	0
1JPP_B	0	2	3	2		1	3	2	1	2	5	0	0
1MQ8_B	0	0	0	1		0	0	0	1	0	0	0	1
1NFI_F	1	0	1	0		1	1	0	0	1	1	0	0
1NUN_A	0	2	1	0		0	2	1	0	0	2	1	0
1UB4_C	0	1	0	0		0	1	0	0	0	1	0	0
2HHB_B	0	0	1	0		0	0	1	0	0	0	1	0

**Table 6:** Performance comparison of existing approaches on the independent test dataset (BID18).

Methods	Classifier		SPE	SEN	PRE	ACC	F1	MCC
HEP	SVM		0.76	0.6	0.84	0.79	0.70	0.56
PredHS-SVM	SVM		0.93	0.79	0.59	0.83	0.68	0.57
iPPHOT	SVM		0.586	0.462	0.794	0.650	0.584	0.353
KFC2a	SVM		0.73	0.55	0.74	0.73	0.63	0.44
KFC2b	SVM		0.87	0.64	0.55	0.77	0.60	0.44
PCRPi	Bayesian network		0.75	0.51	0.39	0.69	0.44	0.25
MINERVA	SVM		0.90	0.65	0.44	0.76	0.52	0.38
APIS	SVM		0.76	0.57	0.72	0.75	0.64	0.45
KFC	Decision trees		0.85	0.48	0.31	0.69	0.38	0.19
Robetta	Knowledge-based method		0.88	0.52	0.33	0.72	0.41	0.25
FOLDEF	Knowledge-based method		0.88	0.48	0.26	0.69	0.34	0.17

## References

[1] Zeng J., Li D., Wu Y., Zou Q., Liu X. (2016). An empirical study of features fusion techniques for protein-protein interaction prediction. Curr. Bioinform..

[2] Moreira I.S., Fernandes P.A., Ramos M.J. (2007). Hot spots—A review of the protein–protein interface determinant amino-acid residues. Proteins Struct. Funct. Bioinform..

[3] Xia J., Yue Z., Di Y., Zhu X., Zheng C.H. (2016). Predicting hot spots in protein interfaces based on protrusion index, pseudohydrophobicityandelectron-ioninteractionpseudopotentialfeatures. Oncotarget.

[4] Thorn K.S., Bogan A.A. (2001). ASEdb: A database of alanine mutations and their effects on the free energy of binding in protein interactions. Bioinformatics.

[5] Fischer T., Arunachalam K., Bailey D., Mangual V., Bakhru S., Russo R., Huang D., Paczkowski M., Lalchandani V., Ramachandra C. (2003). The binding interface database (BID): a compilation of amino acid hot spots in protein interfaces. Bioinformatics.

[6] Kumar M.S., Gromiha M.M. (2006). PINT: protein–protein interactions thermodynamic database. Nucleic Acids Res..

[7] Moal I.H., Fernández-Recio J. (2012). SKEMPI: A Structural Kinetic and Energetic database of Mutant Protein Interactions and its use in empirical models. Bioinformatics.

[8] Li X., Keskin O., Ma B., Nussinov R., Liang J. (2004). Protein-Protein Interactions: Hot Spots and Structurally Conserved Residues often Locate in Complemented Pockets that Pre-organized in the Unbound States: Implications for Docking. J. Mol. Boil..

[9] Clackson T., Wells J.A. (1995). A hot spot of binding energy in a hormone-receptor interface. Science.

[10] Li J., Liu Q. (2009). ‘Double water exclusion’: A hypothesis refining the O-ring theory for the hot spots at protein interfaces. Bioinformatics.

[11] Deng L., Guan J., Wei X., Yi Y., Zhang Q.C., Zhou S. (2013). Boosting prediction performance of protein-protein interaction hot spots by using structural neighborhood properties. J. Comput. Biol..

[12] Deng L., Guan J., Dong Q., Zhou S. (2009). Prediction of protein-protein interaction sites using an ensemble method. BMC Bioinform..

[13] Deng L., Fan C., Zeng Z. (2017). A sparse autoencoder-based deep neural network for protein solvent accessibility and contact number prediction. BMC Bioinform..

[14] Kawashima S., Pokarowski P., Pokarowska M., Kolinski A., Katayama T., Kanehisa M. (2007). AAindex: Amino acid index database, progress report 2008. Nucleic Acids Res..

[15] Chen P., Li J., Wong L., Kuwahara H., Huang J.Z., Gao X. (2013). Accurate prediction of hot spot residues through physicochemical characteristics of amino acid sequences. Proteins Struct. Funct. Bioinform..

[16] Jiang J., Wang N., Chen P., Zheng C., Wang B. (2017). Prediction of Protein Hotspots from Whole Protein Sequences by a Random Projection Ensemble System. Int. J. Mol. Sci..

[17] Altschul S.F., Madden T.L., Schäffer A.A., Zhang J., Zhang Z., Miller W., Lipman D.J. (1997). Gapped BLAST and PSI-BLAST: A New Generation of Protein Database Search Programs. Nucleic Acids Res..

[18] Melo R., Fieldhouse R., Melo A., Correia J.D., Cordeiro M.N.D., Gümüş Z.H., Costa J., Bonvin A.M., Moreira I.S. (2016). A machine learning approach for hot-spot detection at protein-protein interfaces. Int. J. Mol. Sci..

[19] Moreira I.S., Koukos P.I., Melo R., Almeida J.G., Preto A.J., Schaarschmidt J., Trellet M., Gümüs Z.H., Costa J., Bonvin A.M. (2017). SpotOn: High Accuracy Identification of Protein-Protein Interface Hot-Spots. Sci. Rep..

[20] Chan C.H., Liang H.K., Hsiao N.W., Ko M.T., Lyu P.C., Hwang J.K. (2004). Relationship between local structural entropy and protein thermostabilty. Proteins Struct. Funct. Bioinform..

[21] Pan Y., Wang Z., Zhan W., Deng L. (2017). Computational identification of binding energy hot spots in protein-RNA complexes using an ensemble approach. Bioinformatics.

[22] Ashkenazy H., Erez E., Martz E., Pupko T., Ben-Tal N. (2010). ConSurf 2010: calculating evolutionary conservation in sequence and structure of proteins and nucleic acids. Nucleic Acids Res..

[23] Higa R.H., Tozzi C.L. (2009). Prediction of binding hot spot residues by using structural and evolutionary parameters. Genet. Mol. Boil..

[24] Shingate P., Manoharan M., Sukhwal A., Sowdhamini R. (2014). ECMIS: computational approach for the identification of hotspots at protein-protein interfaces. BMC Bioinform..

[25] Joosten R.P., Te Beek T.A., Krieger E., Hekkelman M.L., Hooft R.W., Schneider R., Sander C., Vriend G. (2010). A series of PDB related databases for everyday needs. Nucleic Acids Res..

[26] Lee B., Richards F.M. (1971). The interpretation of protein structures: estimation of static accessibility. J. Mol. Boil..

[27] Tuncbag N., Gursoy A., Keskin O. (2009). Identification of computational hot spots in protein interfaces: combining solvent accessibility and inter-residue potentials improves the accuracy. Bioinformatics.

[28] Xia J.F., Zhao X.M., Song J., Huang D.S. (2010). APIS: accurate prediction of hot spots in protein interfaces by combining protrusion index with solvent accessibility. BMC Bioinform..

[29] Keskin O., Ma B., Nussinov R. (2005). Hot regions in protein–protein interactions: the organization and contribution of structurally conserved hot spot residues. J. Mol. Boil..

[30] Cho K.i., Kim D., Lee D. (2009). A feature-based approach to modeling protein–protein interaction hot spots. Nucleic Acids Res..

[31] Darnell S.J., Page D., Mitchell J.C. (2007). An automated decision-tree approach to predicting protein interaction hot spots. Proteins Struct. Funct. Bioinform..

[32] Liang S., Grishin N.V. (2004). Effective scoring function for protein sequence design. Proteins Struct. Funct. Bioinform..

[33] Lee D.T., Schachter B.J. (1980). Two algorithms for constructing a Delaunay triangulation. Int. J. Comput. Inf. Sci..

[34] Deng L., Zhang Q.C., Chen Z., Meng Y., Guan J., Zhou S. (2014). PredHS: A web server for predicting protein–protein interaction hot spots by using structural neighborhood properties. Nucleic Acids Res..

[35] Kortemme T., Kim D.E., Baker D. (2004). Computational alanine scanning of protein-protein interfaces. Sci. STKE.

[36] Tuncbag N., Keskin O., Gursoy A. (2010). HotPoint: Hot spot prediction server for protein interfaces. Nucleic Acids Res..

[37] Lise S., Archambeau C., Pontil M., Jones D.T. (2009). Prediction of hot spot residues at protein-protein interfaces by combining machine learning and energy-based methods. BMC Bioinform..

[38] Lise S., Buchan D., Pontil M., Jones D.T. (2011). Predictions of hot spot residues at protein-protein interfaces using support vector machines. PLoS ONE.

[39] Liang S., Meroueh S.O., Wang G., Qiu C., Zhou Y. (2009). Consensus scoring for enriching near-native structures from protein–protein docking decoys. Proteins Struct. Funct. Bioinform..

[40] Saeys Y., Inza I., Larrañaga P. (2007). A review of feature selection techniques in bioinformatics. Bioinformatics.

[41] Chen Y.W., Lin C.J. (2006). Combining SVMs with various feature selection strategies. Feature Extraction.

[42] Breiman L. (2001). Random forests. Mach. Learn..

[43] Guyon I., Weston J., Barnhill S., Vapnik V. (2002). Gene selection for cancer classification using support vector machines. Mach. Learn..

[44] Peng H., Long F., Ding C. (2005). Feature selection based on mutual information criteria of max-dependency, max-relevance, and min-redundancy. IEEE Trans. Pattern Anal. Mach. Intell..

[45] Wang S.P., Zhang Q., Lu J., Cai Y.D. (2018). Analysis and prediction of nitrated tyrosine sites with the mRMR method and support vector machine algorithm. Curr. Bioinform..

[46] Zou Q., Zeng J., Cao L., Ji R. (2016). A novel features ranking metric with application to scalable visual and bioinformatics data classification. Neurocomputing.

[47] Wang L., Zhang W., Gao Q., Xiong C. (2014). Prediction of hot spots in protein interfaces using extreme learning machines with the information of spatial neighbour residues. IET Syst. Boil..

[48] Qiao Y., Xiong Y., Gao H., Zhu X., Chen P. (2018). Protein-protein interface hot spots prediction based on a hybrid feature selection strategy. BMC Bioinform..

[49] Wold S., Esbensen K., Geladi P. (1987). Principal component analysis. Chemom. Intell. Lab. Syst..

[50] Jia C., Zuo Y., Zou Q. (2018). O-GlcNAcPRED-II: An integrated classification algorithm for identifying O-GlcNAcylation sites based on fuzzy undersampling and a K-means PCA oversampling technique. Bioinformatics.

[51] Mika S., Ratsch G., Weston J., Scholkopf B., Mullers K.R. (1999). Fisher discriminant analysis with kernels. Neural networks for signal processing IX, 1999. Proceedings of the 1999 IEEE Signal Processing Society Workshop.

[52] Cover T.M. (1967). Nearest Neighbour Pattern Classification. IEEE Trans. Inf. Theory.

[53] Cortes C., Vapnik V. (1995). Support-vector networks. Mach. Learn..

[54] Quinlan J.R. (1986). Induction on decision tree. Mach. Learn..

[55] Friedman N., Dan G., Goldszmidt M. (1997). Bayesian Network Classifiers. Mach. Learn..

[56] Yao X. (1999). Evolving artificial neural networks. Proc. IEEE.

[57] Wan S., Duan Y., Zou Q. (2017). HPSLPred: An ensemble multi-label classifier for human protein subcellular location prediction with imbalanced source. Proteomics.

[58] Hu S.S., Chen P., Wang B., Li J. (2017). Protein binding hot spots prediction from sequence only by a new ensemble learning method. Amino Acids.

[59] Ye L., Kuang Q., Jiang L., Luo J., Jiang Y., Ding Z., Li Y., Li M. (2014). Prediction of hot spots residues in protein–protein interface using network feature and microenvironment feature. Chemom. Intell. Lab. Syst..

[60] Zhu X., Mitchell J.C. (2011). KFC2: A knowledge-based hot spot prediction method based on interface solvation, atomic density, and plasticity features. Proteins Struct. Funct. Bioinform..

[61] Quinlan J.R. (2014). C4. 5: Programs for Machine Learning.

[62] Andersen S.K. (1991). Judea Pearl, Probabilistic Reasoning in Intelligent Systems: Networks of Plausible Inference. Artif. Intell..

[63] Irwin M. (1998). Learning in Graphical Models.

[64] Domingos P., Pazzani M. (1997). On the Optimality of the Simple Bayesian Classifier under Zero-One Loss.

[65] Assi S.A., Tanaka T., Rabbitts T.H., Fernandezfuentes N. (2010). PCRPi: Presaging Critical Residues in Protein interfaces, a new computational tool to chart hot spots in protein interfaces. Nucleic Acids Res..

[66] Ofran Y., Rost B. (2007). Protein-protein interaction hotspots carved into sequences. PLoS Comput. Boil..

[67] Liaw A., Wiener M. (2002). Classification and regression by randomForest. R News.

[68] Freund Y., Schapire R.E. (1997). A decision-theoretic generalization of on-line learning and an application to boosting. J. Comput. Syst. Sci..

[69] Friedman J.H. (2001). Greedy function approximation: A gradient boosting machine. Ann. Stat..

[70] Chen T., Guestrin C. Xgboost: A scalable tree boosting system. Proceedings of the 22nd Acm sigkdd International Conference on Knowledge Discovery and Data Mining.

[71] Wang L., Liu Z.P., Zhang X.S., Chen L. (2012). Prediction of hot spots in protein interfaces using a random forest model with hybrid features. Protein Eng. Des. Sel..

[72] Huang Q., Zhang X. An improved ensemble learning method with SMOTE for protein interaction hot spots prediction. Proceedings of the IEEE International Conference on Bioinformatics and Biomedicine.

[73] Chawla N.V., Bowyer K.W., Hall L.O., Kegelmeyer W.P. (2002). SMOTE: synthetic minority over-sampling technique. J. Artif. Intell. Res..

[74] Petukh M., Li M., Alexov E. (2015). Predicting binding free energy change caused by point mutations with knowledge-modified MM/PBSA method. PLoS Comput. Biol..

[75] Li W., Godzik A. (2006). Cd-hit: A fast program for clustering and comparing large sets of protein or nucleotide sequences. Bioinformatics.

[76] Henikoff S., Henikoff J.G. (1992). Amino acid substitution matrices from protein blocks. Proc. Natl. Acad. Sci. USA.

[77] Rost B., Sander C. (1994). Conservation and prediction of solvent accessibility in protein families. Proteins Struct. Funct. Bioinform..

[78] Hamelryck T. (2005). An amino acid has two sides: a new 2D measure provides a different view of solvent exposure. Proteins Struct. Funct. Bioinform..

[79] Segura M.J., Assi S.A., Fernandez-Fuentes N. (2010). Presaging critical residues in protein interfaces-web server (PCRPi-W): a web server to chart hot spots in protein interfaces. PLoS ONE.

[80] Kortemme T., Baker D. (2002). A simple physical model for binding energy hot spots in protein–protein complexes. Proc. Natl. Acad. Sci. USA.

[81] Guerois R., Nielsen J.E., Serrano L. (2002). Predicting changes in the stability of proteins and protein complexes: A study of more than 1000 mutations. J. Mol. Boil..

